# Description of Neuropsychological Profile in Patients with 22q11 Syndrome

**DOI:** 10.3390/genes14071347

**Published:** 2023-06-26

**Authors:** Joga-Elvira Lorena, Palma-Robleda Sandra

**Affiliations:** 1 Pediatria, Parc Tauli Hospital Universitari, Institut d’Investigació i Innovació Parc Tauli (I3PT-CERCA), 08208 Sabadell, Spain; 2 Universitat de Barcelona, 08007 Barcelona, Spain

**Keywords:** 22q11.2 syndrome, DiGeorge syndrome, velocardiofacial syndrome, rare disease, cognition, neuropsychology

## Abstract

Background: 22q11 deletion syndrome (SD22Q11) is a neurogenetic condition that is associated with a high risk of neurodevelopmental disorders and intellectual disability. People with SD22Q11, both children and adults, often experience significant difficulties in social interactions, as well as neurocognitive deficits, and have elevated rates of autism spectrum disorder (ASD). Despite this, the relationship between basic cognitive processes and cognitive ability in this population has not been well investigated. Methods: the main objective of the present research is to describe the neurocognitive profile of people with SD22Q11 using standardized neuropsychological assessment instruments. For this purpose, a sample of 10 participants aged between 7 and 15 years was administered an assessment battery with the following tests: WISC-V, CELF-5, NEPSY-II, CSAT-R, CARAS-R, TP, MABC-2, BRIEF-2, SENA, DABAS, ABAS-II, SCQ, and ADOS-2. Results: the results showed IQ scores in the borderline normal range, as well as difficulties in language functions, social skills, motor skills, and executive functions. Conclusions: an individualized assessment taking into account the globality of its expression, and a therapeutic approach adapted to the specific needs of children with this syndrome is essential.

## 1. Introduction

SD22Q11 deletion syndrome, also known as “DiGeorge syndrome” and/or “velocardiofacial syndrome” is a rare disease caused by a deletion on chromosome 22, specifically at the q11.2 locus [1]. SD22Q11 deletion syndrome is the most common of all rare syndromes affecting the same chromosomal region. It affects 1 in 4000 live newborns [2,3,4,5,6,7] and its incidence is equal for both sexes [8].

Individuals with SD22Q11 often have developmental delays [8,9,10]. In most cases, IQ is in the range of mild intellectual disability or borderline [11]. On the other hand, several studies have noted a variety of associated cognitive deficits, in particular, difficulties in attention, executive functioning (the most prominent being working memory), visuospatial processing, arithmetic, and sensorimotor skills [12,13,14,15,16,17,18,19,20]. 

Commonly, cognitive impairments translate into significant difficulties in school learning and functional adaptive life skills, which may affect the person’s socioemotional functioning. 

In recent years, several studies have measured whether there are deficits in behavior and social cognition in people with SD22Q11, concluding that young people with SD22Q11 have difficulties in initiating and maintaining social relationships [21,22]. 

However, although there are descriptions of partial neuropsychological profiles in the pediatric population, to our knowledge, no published study to date has investigated in the Spanish population the differences between the social cognition profiles of individuals with a highly pervasive genetic condition such as SD22Q11. Therefore, examining social cognition and its relationship with global intellectual function and social behavior in SD22Q11 may facilitate the development of more specific treatment options and, as a result, improve their quality of life. 

With increasing advances in behavioral genetics, it has become possible to identify more precisely the connections between specific genetic or chromosomal disorders and the unique neurocognitive and behavioral characteristics of affected individuals. In this context, the present research will focus on a neuropsychological assessment of cognitive functions that have been less studied to date. Thus, the aims of this study are twofold: to describe what strengths and weaknesses children with SD22Q11 share, and to quantify the proportion of children with SD22Q11 at risk for bullying.

## 2. Materials and Methods

### 2.1. Sample

This is a descriptive study with a trial sample, as SD22Q11 encompasses a nonrandom type of population with specific characteristics.

The inclusion criteria were the presence of a genetically confirmed 22Q11 deletion using the array comparative genomic hybridization (aCGH) test, age between 7 and 16 years old, and Spanish mother tongue. The evaluation protocol was approved by the Ethics Committee of the CSPT, specifically the Research Ethics Committee (CEI) of Parc Taulí (2022/5093). Written informed consent was obtained from all parents or legal guardians and children over 12 years of age. 

All participants were recruited through voluntary response sampling techniques by sending information leaflets to the 22Q11 association, as well as with the collaboration of patients treated at the Consorci Corporació Sanitària Parc Taulí de Sabadell (CCSPT).

### 2.2. Measuring Instruments

Array comparative genomic hybridization (aCGH) is a molecular technique used to detect chromosomal imbalances, including deletions and duplications, throughout the entire genome. In aCGH, DNA from the patient and a reference sample are labeled with different fluorescent dyes, and then cohybridized to an array containing thousands or millions of DNA probes representing specific genomic regions. The relative intensities of the fluorescent signals are compared, and any differences indicate the presence of genomic imbalances, including the 22q11.2 deletion. aCGH provides high-resolution detection of copy number variations, and is commonly used in clinical genetics to diagnose genetic disorders and identify chromosomal abnormalities.

When carrying out the neurocognitive assessment, various standardized tools and tests were used to ensure the validity and reliability of the obtained results.

First, the Wechsler Intelligence Scale for Children, Fifth Edition (WISC-V) for ages 7:6 to 16:11 years was administered to measure the IQ of the participants (X = 100 ± 15). The necessary scales were used to obtain the corresponding primary and secondary indices [23].

In addition, two standardized assessment instruments were used to measure participants’ language: The Clinical Evaluation of Language Fundamentals, Fifth Edition (CELF-5) (X = 100 ± 15), more specifically the sentence elaboration subtests and pragmatic skills profile [24], and the Neuropsychological Battery for Children, Second Edition (NEPSY-II) (2007) (X = 100 ± 15), focusing on the semantic verbal fluency and comprehension of instructions subtests [25]. These instruments allow for a comprehensive and detailed measurement of different aspects of language, such as grammatical production, pragmatics, and semantic comprehension. 

To measure executive functions, the Behavioral Assessment of Executive Function, Second Edition (BRIEF-2) tool was used; this is a test designed to assess everyday and behavioral aspects of executive functions by parents and teachers (X = 50 ± 10) [26].

In addition, selective/sustained attention was measured using different tests specific to each age range depending on the profile, such as the Perception of Differences Test, Revised (Faces-R) (X = 100 ± 15) [27], the Toulouse–Pieron Test, Revised (TP-R) (X = 100 ± 15) [28], and the Childhood Sustained Attention Task, Revised (C-SAT) (X = 100 ± 15) [29].

To measure social cognition, the Autism Diagnostic Observation Scale, Second Edition (ADOS-2) was used to provide a comprehensive assessment of communication, social interaction, and play or imaginative use of materials [30].

Regarding motor development in children aged 3–16 years old, the Movement Assessment Battery for Children, Second Edition (MABC-2) was used, which allows the assessment of not only fine motor skills, but also other aspects related to motor coordination, manual dexterity, balance, agility, speed, and coordination of the upper limbs, as well as strength (X = 100 ± 15) [31].

Three questionnaires were used to assess participants’ behavior: The Child and Adolescent Assessment System (SENA), which is a behavioral screening test (X = 50 ± 10) [32], the Attention Deficit Hyperactivity Disorder Rating Scale (ADHD Rating Scale) [33], and the Social Communication Questionnaire (SCQ) [34]. The latter, the SCQ, is a 40-question parent-completed ASD screening tool on communication and social functioning with similar content to the ADI-R, with the correlation between the ADI-R total score and the SCQ total score being 0.71 [35].

Finally, two questionnaires were used to measure adaptive behavior in different settings: the Diagnostic Adaptive Behavioral Scale (DABS) for the family (X = 100 ± 15) [36], and the Adaptive Behavior Assessment System (ABAS-II) for the school (X = 100 ± 15) [37].

Of the analyzed sample, not all participants completed the neuropsychological assessment in its entirety. In this sense, the Wechsler intelligence scales were completed by all participants. In contrast, the NEPSY-II was completed by 9 of the 10 participants, as was the case with the attention tests. The MABC-2, CELF-5, and ADOS-2 tests were completed by 7 of the 10 participants.

The direct scores for all the cognitive and adaptive functioning test were converted into standard scores with a mean of 100 and a standard deviation of 15. The direct scores for the SENA and BRIEF-2 were converted into standard scores with a mean of 50 and standard deviation of 10, and the direct scores for the ADHD Rating Scale were converted into percentile scores.

#### Statistical Analysis

The basic demographic characteristics of the patients (sex and age) and the scores of the tests administered in comparison with the current Spanish scale were analyzed with R version 1.1.456^®^ [38]. As this was a descriptive study, boxplots, also known as box-and-whisker plots, are a visual representation showing the distribution of a set of numerical data.

## 3. Results

**Figure 1:**
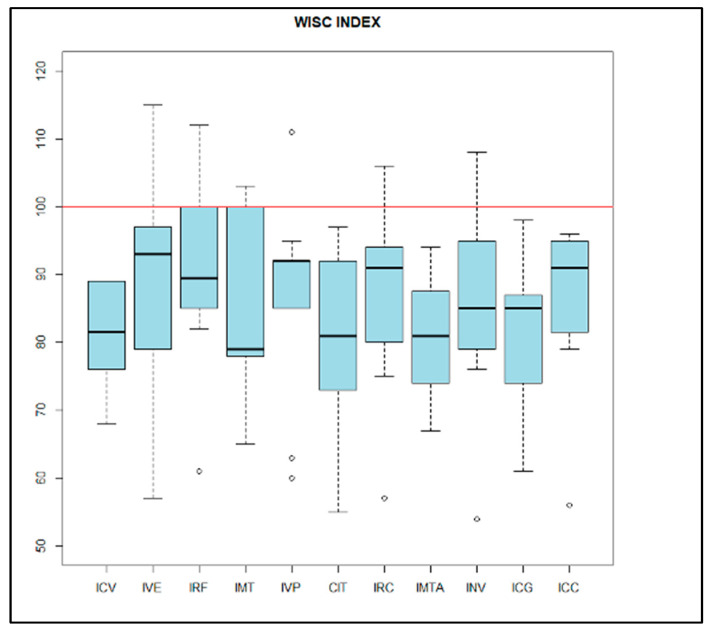
WISC-V SCALES. ICV: verbal comprehension index. IVE: visuospatial index. IRF: index of fluid reasoning. IMT: working memory index. IVP: index of processing speed. CIT: total intellectual quotient. IRC: quantitative reasoning index. IMTA: auditory working memory index. INV: nonverbal reasoning index. ICG: general capacity index. ICC: cognitive competence index.

**Figure 2:**
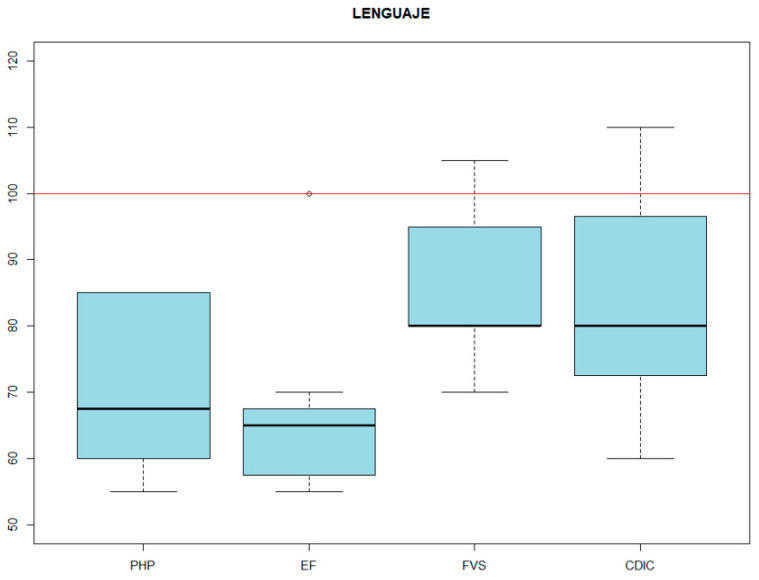
PHP LANGUAGE VARIABLES. Pragmatic skills profile. EF: sentence elaboration. FVS: semantic verbal fluency. CDIC: comprehension of instructions.

The results of 10 patients who met the above inclusion criteria were reviewed. The mean age was 10.40 years old, with a standard deviation (SD) of 3.16 years. Females accounted for 30% of the sample, and males for 70%. With regard to the neuropsychological analysis, deficits were found in all cognitive domains analyzed (Figure 1). The mean IQ was 80.6 (SD = 12.48). Significant deficits were also found in language, both receptive and expressive, along with pragmatic skills (Figure 2). Attention showed a very heterogeneous pattern among subjects, with a mean of 85.78 and a standard deviation of 25.34. 

**Figure 3:**
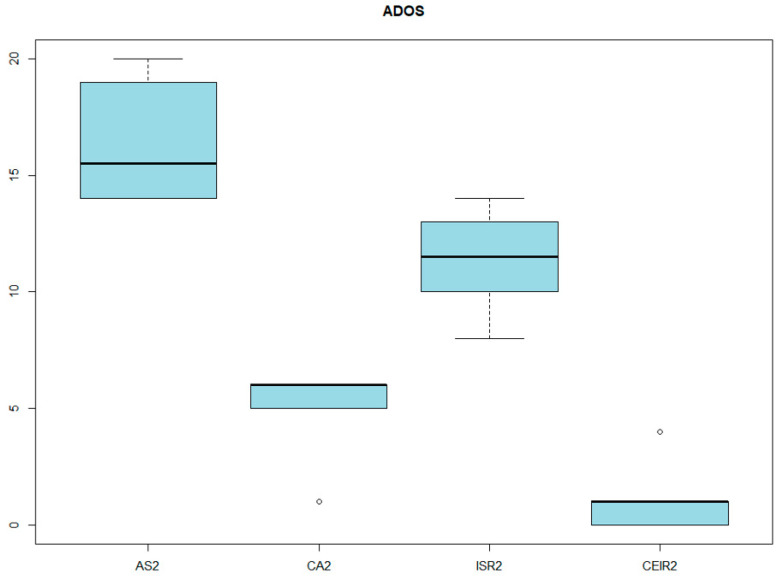
ADOS-2 RESULTS. AS2: social affectation. CA2: communication. ISR2: reciprocal social interaction. CEIR2: stereotyped behaviors and restricted interests.

From all of the data collected, it was observed that patients who were administered the ADOS-2 scored significantly on the total test. However, with regard to the stereotyped behaviors and restricted interests section, only one patient showed a high score, while the rest did not show significant scores in this section. In relation to social affect, communication, and reciprocal social interaction, all subjects obtained high scores (Figure 3). The total mean of this test was 16.33 points with an SD of 2.58 points. Continuing with the assessment of social cognition, the SCQ was administered and half of the participants were found to score high on both forms (Form A, lifetime, and Form B, current), whereby the caregivers determined the possible presence of autistic spectrum disorders.

**Figure 4:**
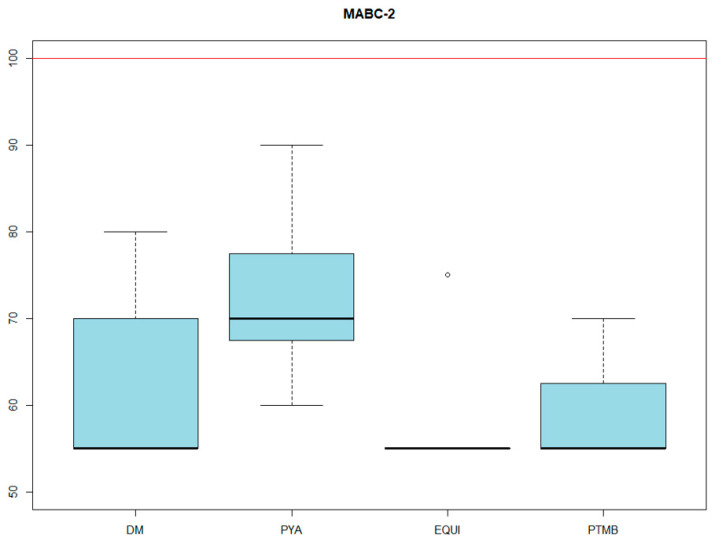
MABC-2 RESULTS. DM: manual dexterity score. PYA: pointing and catching score. EQUI: balance. PTMB: total score.

With specific regard to both fine and gross motor skills, in addition to balance, all had severe difficulties, with a total test mean of 59.28 with an SD of 7.31 (Figure 4).

**Figure 5:**
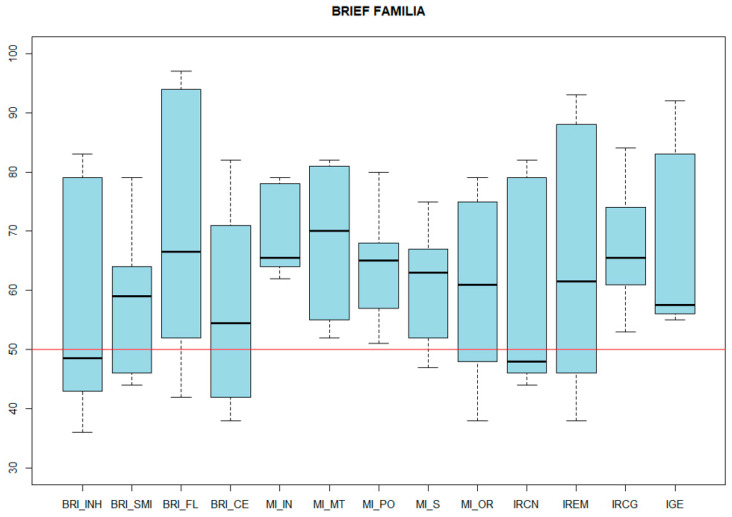
BRIEF-2 RESULTS (family). BRI_INH: inhibition. BRI_SMI: supervision. BRI_FL: flexibility. BRI_CE: emotional control. MI_IN: initiative. MI_MT: working memory. MI_PO: planning-organization. MI_S: supervision. MI_OR: material organization. IRCN: index of behavioral regulation. IREM: emotional regulation index. IRCG: index of cognitive regulation. IGE: global index of executive function.

**Figure 6:**
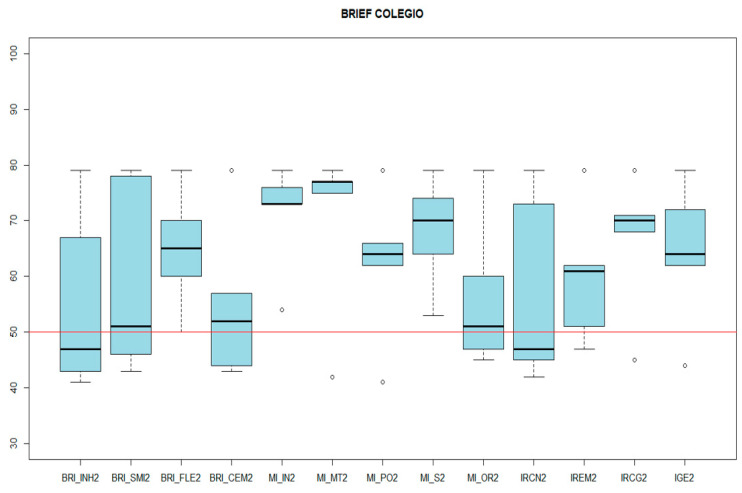
BRIEF-2 RESULTS (school). BRI_INH2: inhibition. BRI_SMI2: supervision. BRI_FLE2: flexibility. BRI_CEM2: emotional control. MI_IN2: initiative. MI_MT2: working memory. MI_PO2: planning-organization. MI_S2: supervision. MI_OR2: material organization. IRCN2: index of behavioral regulation. IREM2: emotional regulation index. IRCG2: index of cognitive regulation. IGE2: global index of executive function.

In the questionnaires given to the family and the school, a concordance was found between systems between adaptive behavior and executive functions. In the case of the BRIEF-2, both in the school itself and in the Behavioral Regulation Index, it was the most preserved, with the mean at home being 57.2 ± 17.38, and the mean at school being 60.2 ± 18.68. In contrast, the index with the greatest impairment was the Cognitive Regulation Index, which reports the degree of difficulty of the person assessed to control and manage their cognitive processes and solve problems effectively. The results of the Emotional Regulation Index stand out, which vary up to 10 points depending on the context, with the mean in the family environment being 70 (SD 19.98), and 60 (SD 12.40) at school (Figure 5 and Figure 6, respectively).

Regarding the SENA, total coincidence of scores was found in both contexts: as a weak point, both highlight the Emotional Problems Index, with a mean score of 72.75 (SD 8.56) at home and 66.71 (SD 15.16) at school. On the other hand, as a strong point, both coincide in the Personal Resources Index, this index being in the normal range, with 33.42 (SD 9.18) at school and 33.66 (SD 12) at home.

**Figure 7:**
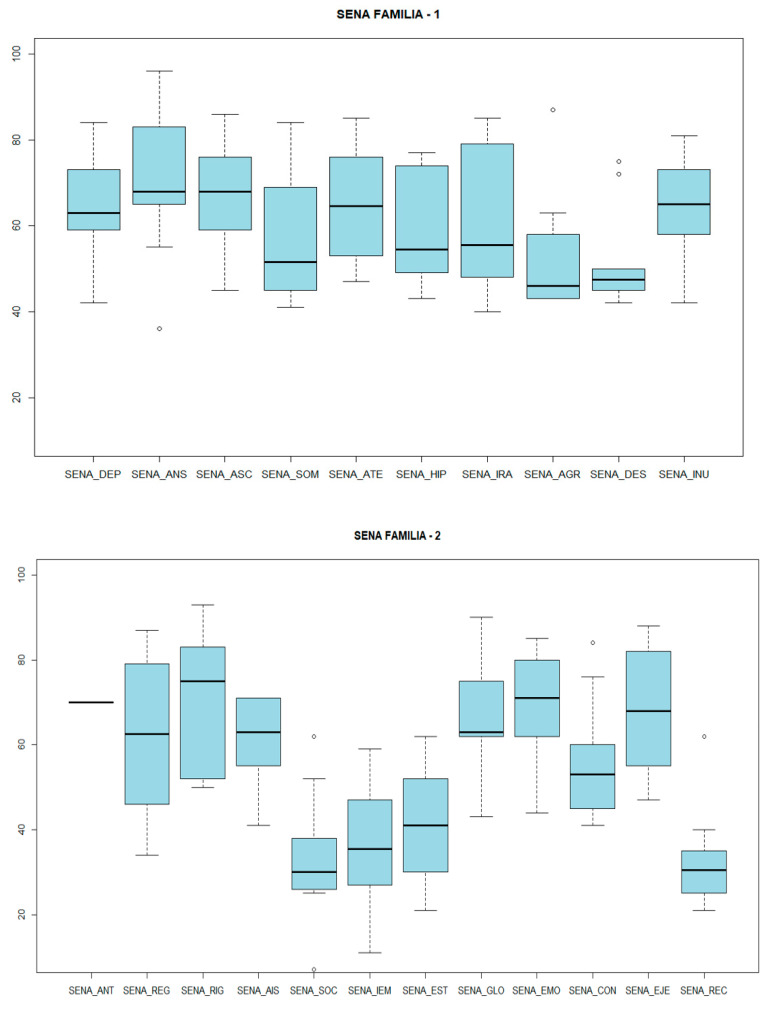
SENA RESULTS (family). SENA_DEP: depression. SENA_ANS: anxiety. SENA_ASC: social anxiety. SENA_SOM: somatic complaints. SENA_ATE: attention problems. SENA_HIP: hyperactivity-impulsivity. SENA_IRA: anger control problems. SENA_AGR: aggression. SENA_DES: defiant behavior. SENA_ANT: antisocial behavior. SENA_INU: unusual behavior. SENA_REG: emotional regulation problems. SENA_RIG: rigidity. SENA_AIS: isolation. SENA_SOC: integration and social competence. SENA_IEM: emotional intelligence. SENA_EST: willingness to study. SENA_GLO: global problem index. SENA_EMO: emotional problems index. SENA_CON: behavioral problems index. SENA_EJE: index of executive functioning problems. SENA_REC: index of personal resources.

**Figure 8:**
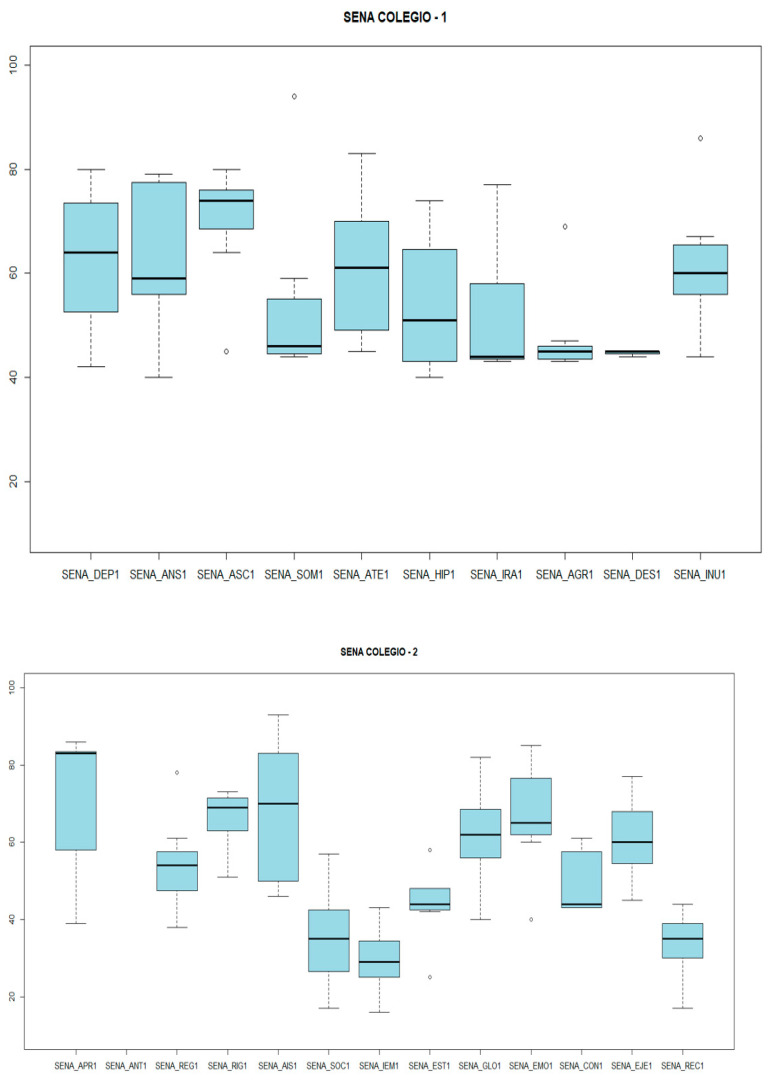
SENA RESULTS (School). SENA_DEP1: depression. SENA_ANS1: anxiety. SENA_ASC1: social anxiety. SENA_SOM1: somatic complaints. SENA_ATE1: attention problems. SENA_HIP1: hyperactivity-impulsivity. SENA_IRA1: anger control problems. SENA_AGR1: aggression. SENA_DES1: defiant behavior. SENA_ANT1: antisocial behavior. SENA_INU1: unusual behavior. SENA_APR1: learning disabilities. SENA_REG1: emotional regulation problems. SENA_RIG1: rigidity. SENA_AIS1: isolation. SENA_SOC1: integration and social competence. SENA_IEM1: emotional intelligence. SENA_EST1: willingness to study. SENA_GLO1: global index of problems. SENA_EMO1: index of emotional problems. SENA_CON1: index of behavioral problems. SENA_EJE1: index of executive functioning problems. SENA_REC1: index of personal resources.

Another aspect analyzed was the presence of bullying based on the critical items of the SENA: “Risk of bullying”, “Lack of social support”, “Isolation from peers”, “Insults from peers”, and “Fear of a peer”. In the sample, it was found that 33% of the cases reported by parents and 42% reported by the school presented a risk of bullying, and the school reported that 71.4% of the cases presented a lack of social support. With regard to isolation and insult from peers, the family reported 22% of both. The school reported 28% and 14%, respectively. Finally, only one family reported fear of a peer (14%) (Figure 7 and Figure 8). As the figures above show, clinically significant levels of anxiety/depression, unusual behavior, attention problems, inhibition, somatic complaints, and social problems were identified within the sample. However, clinical scales for externalizing behaviors, such as challenging behavior problems and aggression, were found to be in the normal range.

Further to the description, 44% of the sample showed signs of attention deficit hyperactivity disorder (ADHD), and 22% scored in the borderline range. Of these, only 14% were also reported by the school at full significance, and 28% at borderline significance. 

**Figure 9:**
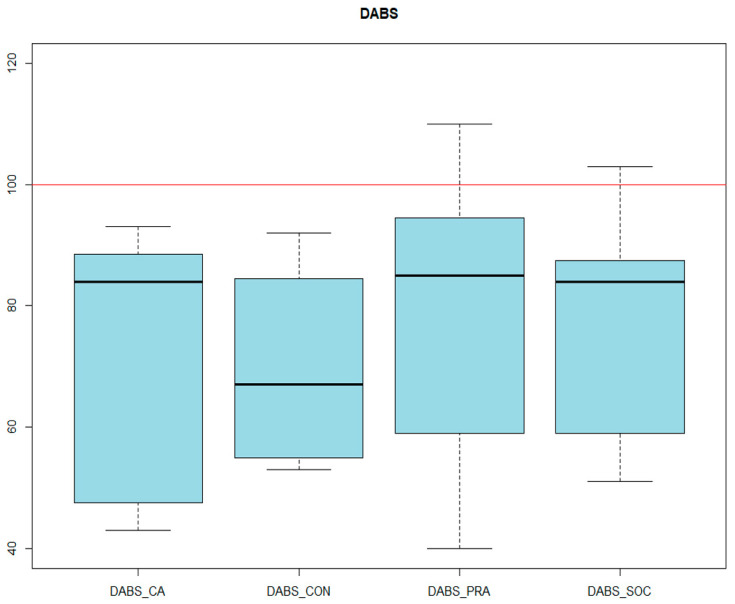
DABS RESULTS. DABS_CA: adaptive behavior index. DABS_CON: conceptual. DABS_SOC: social. DABS_PRA: practical.

**Figure 10:**
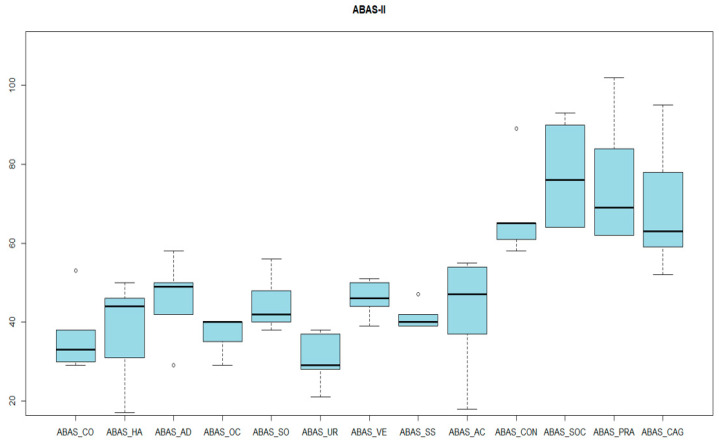
ABAS-II RESULTS. ABAS_CO: communication. ABAS_HA: social skills. ABAS_AD: self-direction. ABAS_OC: leisure. ABAS_SO: social. ABAS_UR: use of community resources. ABAS_VE: school life. ABAS_SS: health and safety. ABAS_AC: self-care. ABAS_CON: conceptual index. ABAS_SOC: social index. ABAS_PRA: practical index. ABAS_CAG: general adaptive behavior index.

Finally, as far as adaptive behavior is concerned, in the DABS, all scored in the range of significance, the Adaptive Behavior Index being 68 ± 24 (Figure 8). In parallel, in the ABAS-II, the mean score of the General Adaptive Behavior Index was 69.4 ± 17.18 (Figure 9 and Figure 10).

## 4. Discussion

This study focuses on conducting a neuropsychological study to explore functions that have not been extensively studied so far. The aim was to describe common strengths and weaknesses in boys and girls with this chromosomal syndrome.

22Q11 syndrome is characterized by an unusual neuropsychological profile in multiple areas, with global cognitive performance at the borderline of normal, with verbal IQ significantly lower than nonverbal performance. Impairments in executive functions, academic skills (language and mathematics), motor skills, and social cognition are also observed.

The literature, on the contrary, exposes that they show better skills in verbal tasks, such as verbal reasoning and auditory comprehension, while they may have difficulties in visuospatial skills [19]; this may have several justifications. The first cause is the distribution of the sample; being a sample composed of 70% male subjects, it is known from the literature and functional magnetic resonance imaging (fMRI) studies that males may perform slightly better in certain tasks related to spatial or visuospatial memory due to a higher activation in certain brain areas, such as the parietal gyrus, during visuospatial memory tasks [39]. Another possible rationale is that more recent studies focus on a broader age group, and patterns of neurocognitive strengths and weaknesses may change with age. Future studies would be needed to accurately characterize the neurocognitive phenotype of children with 22Q11 deletion syndrome as a function of sex and developmental timing.

In relation to the social problems that have been described, to our knowledge, this is one of the few statewide studies reporting a diagnosis of autism spectrum disorder (ASD) in the context of SD22Q11. A clinical approach to diagnosis was used, consisting of a combination of the Autism Observation Scale (ADOS-2) and the Screening Questionnaire for Autism in Childhood (SCQ).

Although it is important to keep in mind that research assessments do not replace clinical assessments, it is useful to examine ASD diagnostic performance using criteria that more closely resemble those used in clinical practice. In the present study, none of the participants strictly met the diagnostic criteria for ASD according to the ADOS-2 and SCQ scores. This is because, despite scoring significantly on the ADOS-2, the presence of repetitive behaviors or restricted interests, which are a key criterion according to the DSM-5 for the diagnosis of ASD, were not recorded or reported by the families [40].

Nevertheless, the results obtained and the literature published to date show that a significant proportion of children with SD22Q11 have impairments in social behavior, theory of mind, and nonverbal communication (e.g., eye contact) [41], and this is especially noticeable when assessing peer relationships. For this reason, the presence of bullying situations was also assessed. Based on the SENA questionnaire, the results showed that 66% of the children were at risk of being bullied. It is therefore essential to study how the social interaction is going, since this reduced social competence in children with SD22Q11 can lead to exclusion or rejection, and can even lead to insults or bullying by their peers. In the studied sample, the school reported that two out of nine subjects were victims of insults from their peers. Difficulties in attention regulation may also contribute to social impairments. Both individuals with ASD and those with SD22Q11 tend to have difficulties in shifting their focus of attention. Problems with joint or shared attention are present in individuals with SD22Q11, regardless of ASD diagnosis [42]

It could be true that many of the social difficulties observed in individuals with SD22Q11 can be explained by a number of factors, including developmental delay, which result in a disadvantage compared to their peers. In addition, anxiety, along with difficulties in maintaining attention and processing emotions, are also contributing factors to these social difficulties [43]. Developmental delay can affect the level of understanding of social norms and interaction skills, making it difficult to adapt to complex social situations. Anxiety can manifest as fear or insecurity in social contexts, preventing active participation and the building of interpersonal relationships. Similarly, problems in maintaining attention and processing emotions can interfere with the ability to read social cues and respond appropriately.

At the level of communication, children with SD22Q11 present significant problems, especially in relation to the use of more complex and abstract language. Communication difficulties have been observed, including poor speech and limitations in language content. Individuals with SD22Q11 have been found to have lower scores in the communication domain, but not in the domains of restricted interests or repetitive behaviors. These results support the idea that communication is an area of weakness in 22Q11 syndrome, especially for those who score in the autistic range according to the ADOS-2 scale.

It is important to mention that the studied sample meets DSM-5 diagnostic criteria [40] for developmental coordination disorder, since the subjects presented difficulties in the acquisition and execution of motor skills, such as inaccuracy in tasks of picking up objects, which were observed in the MABC-2 test. These difficulties could affect activities of daily living, such as their social functioning. Children tend to interact through play, and in many of these games good motor skills are very important, especially at an early age. Difficulties in this domain of development could lead to difficulties in social integration and social competence of children with SD22Q11, but more research is needed. 

Developmental coordination disorder (DCD) is a neurodevelopmental disorder characterized by deficits in motor functions based on what is expected for chronological age, originating in infancy. Another noteworthy fact about this disorder is that it is independent of intelligence; moreover, it is usually associated with neurocognitive deficits and is not explained by any motor or sensory deficit. Similar studies [43] have obtained similar results in terms of motor skills, indicating that all but the earliest motor developmental milestones (e.g., rolling) are later than expected and less valuable than compared to a sample with normotypical development. Furthermore, comparing the results with the only study that has used this battery in children with SD22Q11 [44], we see that 88.9% of the children in the mentioned study presented problems in manual dexterity, balance, and fine motor skills. 

### Limitations

Limitations were encountered during this study that may affect the validity of the results. 

The particular characteristics of the studied population may pose challenges. These characteristics made it difficult to recruit a large, representative, and equitable sample, which affects the generalizability of the results and raises doubts about the statistical significance of the findings.

The attrition experienced in the loss of subjects is another factor to consider. Some participants dropped out of the study due to the length of the proposed sessions or lack of availability. This may have affected the representativeness of the sample and limited the generalizability of the obtained results.

The analyses reported in this study were based on comparisons with the means of the standardization sample, which limits the conclusions that can be drawn. In future studies, it would be additionally informative to compare the performance of children with SD22Q11 with a control sample of children with learning disabilities who do not have SD22Q11.

## 5. Conclusions

22Q11 deletion syndrome has a strong impact on patients’ daily activities due to its clinical symptoms. 

The results show a significant impairment in the area of motor skills (manual dexterity, aiming and catching, balance, etc.) that may affect their daily life.

In addition, although all subjects scored significantly on the ADOS-2, only one of the participants met the criteria for a diagnosis of ASD. All showed impairment in the social domain, but only one showed the presence of stereotyped behaviors and restricted interests. 

However, there are interventions that can improve the social, cognitive, and emotional skills of these individuals, which in turn could improve their autonomy to carry out various activities in their daily lives. 

Based on the described profile, there are some interventions that would be suitable for these subjects, such as: speech therapy (to improve both expressive and receptive language), psycho-pedagogical re-education (to work on memory and attention deficit), physiotherapy (for motor development), and psychotherapy (to improve their social skills).

In addition to performing a more detailed analysis of the syndrome, this study proposes a possible line of future research, working on the application of these interventions to analyze their impact. This could offer a new perspective on the future of SD22Q11.

## Data Availability

Data from this research is available on request.
